# Magnetic Gold Nanoparticle-Labeled Heparanase Monoclonal Antibody and its Subsequent Application for Tumor Magnetic Resonance Imaging

**DOI:** 10.1186/s11671-018-2518-1

**Published:** 2018-04-18

**Authors:** Ning Li, Meng-Meng Jie, Min Yang, Li Tang, Si-Yuan Chen, Xue-Mei Sun, Bo Tang, Shi-Ming Yang

**Affiliations:** 1 Department of Gastroenterology, Xinqiao Hospital, Third Military Medical University (Army Medical University), Chongqing, 400030 China; 2 Department of Gastroenterology, Institute of Surgery research, Daping Hospital, Third Military Medical University (Army Medical University), Chongqing, 400042 China

**Keywords:** Heparanase, Monoclonal antibody, Molecular imaging, Magnetic gold nanoparticle, Magnetic resonance imaging

## Abstract

Heparanase (HPA) is ubiquitously expressed in various metastatic malignant tumors; previous studies have demonstrated that HPA was a potential tumor-associated antigen (TAA) for tumor immunotherapy. We sought to evaluate the feasibility of HPA as a common TAA for magnetic resonance imaging (MRI) of tumor metastasis and its potential application in tumor molecular imaging. We prepared a targeted probe based on magnetic gold nanoparticles coupled with an anti-HPA antibody for the specific detection of HPA by MRI. The specificity of the targeted probe was validated in vitro by incubation of the probe with various tumor cells, and the probe was able to selectively detect HPA (+) cells. We found the probes displayed significantly reduced signal intensity in several tumor cells, and the signal intensity decreased significantly after the targeted probe was injected in tumor-bearing nude mice. In the study, we demonstrated that the HPA&GoldMag probe had excellent physical and chemical properties and immune activities and could specifically target many tumor cell tissues both in vitro and in vivo. This may provide an experimental base for molecular imaging of tumor highly expressing heparanase using HPA mAbs.

## Background

Tumor metastasis is a malignant behavior that is the major cause of death in tumor patients. There are currently no effective strategies for detecting early tumor metastasis. Type-B ultrasounds, computed tomography (CT) scans, and magnetic resonance imaging (MRI) are currently the standard tools for the diagnosis of tumor metastasis, but they can only distinguish metastatic tumors of certain sizes when most patients already suffer distant metastasis [1, 2]. Thus, it is urgent to develop novel strategies to detect early tumor metastasis.

In recent years, molecular imaging, such as MR molecular imaging, has become a new option for the early diagnosis and treatment of tumors because of its good spatial resolution and average contrast agent sensitivity [3, 4]. The development of new contrast agents that provide more than imaging enhancement is one of the main motivations in MRI development. Recently, the use of superparamagnetic molecular probes as a strong signal-amplifying system greatly increases the sensitivity of MR targeting contrast agents. Paramagnetic ion complexes or magnetic particle-conjugated mAbs have been used to change the tumor relaxation time [5]. Molecular imaging using a combination of magnetic nanomaterials and mAbs against tumor-associated antigens (TAAs) is a focus of recent research. Most of the TAAs discovered to date, such as AFP, PSA, and CEA, are tumor tissue-specific [6]. Thus, combining mAbs against these TAAs with magnetic nanomaterials is useful for tumors expressing these specific antigens. Therefore, identification of a common tumor antigen that is suitable for a range of tumors and labeling mAbs against such an antigen with magnetic nanomaterials is useful in tumor molecular imaging.

Heparanase (HPA) is a tumor metastasis-associated gene that was cloned simultaneously by four laboratories in 1999 [7–10]. Only endogenous endoglycosidase can degrade heparan sulfate proteoglycan (HSPG), the main proteoglycan component in the extracellular matrix (ECM). HPA is ubiquitously expressed in metastatic malignant tumors, and its expression level is closely associated with tumor metastasis. HPA can disrupt the integrity of the ECM and basement membrane (BM) through cleavage of HSPG in the ECM and BM, leading to the release and activation of molecules anchored in the ECM and then promoting tumor angiogenesis and metastasis [11, 12]. Our previous studies have demonstrated that HPA can be used as a TAA for tumor immunotherapy [13–16]. Here, we sought to evaluate whether HPA is available to be a common TAA for molecular imaging of tumor metastasis and its potential application in tumor molecular imaging.

In this study, we used magnetic gold nanoparticles (30 nm) as the contrast agent and HPA mAbs as the targeting vectors to construct HPA&GoldMag molecular probes, and we examined the feasibility and significance of these probes in MR molecular imaging for the early diagnosis of tumor metastasis. The in vitro anti-tumor biological effects of HPA antibodies were also evaluated to provide an experimental basis for the application of HPA mAbs in tumor treatment.

## Methods

### Cell Lines and Mice

Seven cell lines were used in this study. Heparanase-positive cell lines, including liver cancer cell line HepG2, human gastric cancer cell lines MKN45 and SGC-7901, colon cancer cell line SW480, and human osteosarcoma cell line U2OS, were purchased from the American Type Culture Collection. Heparanase-negative cell lines, human breast cancer cell line MCF-7, and human primary embryonic fibroblast cell line HF were provided by Dr. Liang (Burn Research Institute, Third Military Medical University, Chongqing, China). HepG2 cells were cultured in DMEM containing 10% fetal bovine serum. SGC-7901, SW480, U2OS, and HF cells were cultured in RPMI 1640 containing 10% fetal bovine serum. MKN45 and MCF-7 cells were cultured in RPMI 1640 containing 15% fetal bovine serum. All cells were cultured in a 5% CO_2_ incubator at 37 °C and were passaged every 24–48 h. Fifteen BALB/c nude mice (4 to 5 weeks old) were purchased from the Animal Department, Third Military Medical University. Animal studies were performed in agreement with the local ethics committee of the Third Military Medical University. All nude mice were maintained in a specific pathogen-free environment.

### Western Blot

Western blot was performed to detect Hpa protein expression following the procedure described in our previous study [17]. Each cell line was lysed with the M-PER extraction reagent (Pierce Co.). Protein concentrations were determined by BCA assay (Bio-Rad, CA, USA). Thirty micrograms of proteins were fractionated by 10% SDS-PAGE and then transferred to a polyvinylidenedifluoride (PVDF) membrane (Roche, Rotkreuz, Switzerland). The membrane was blocked with 5% (*w*/*v*) skim milk in TBST (20 mM Tris-HCl [pH 8.0], 150 mM NaCl, and 0.1% [*v*/*v*] Tween-20) for 2 h at room temperature. Then, the membrane was probed with a 1:200 dilution of anti-HPA antibody (Insight, Israel) in blocking buffer at 4 °C overnight. The membrane was washed four times with TBST and incubated with a horseradish peroxidase-conjugated antibody against mouse IgG for 1 h at room temperature. The membrane was then rinsed with TBST, and the protein bands were visualized with ECL Western Blotting Detection Reagents (GE Healthcare, NJ, USA). The images were analyzed with Quantity One 4.1 software (Bio-Rad). The experiments were repeated at least three times.

### Immunohistochemical Staining

Hpa expression in the above cell lines was detected by immunocytochemistry. Briefly, the above cells were cultured on sterile coverslips at 37 °C in a 5% CO_2_ incubator for 48 h. After endogenous peroxidase activity was blocked by treatment with a 0.3% H_2_O_2_-methanol solution for 20 min, cells were incubated with the Hpa primary antibody (the dilution of the antibody was 1:100) overnight at 4 °C. After thoroughly washing with PBS containing 0.1% Triton X-100, the slides were incubated with a secondary antibody for 20 min at 20 °C. Finally, the slides were incubated for 15 min with an avidin-biotin enzyme reagent. The slides were then immersed into a 3,3′-diaminobenzidine/H2O2 solution. PBS was used as a negative control.

### Construction of HPA&GoldMag Molecular Probe and Atomic Force Micrograph

Construction of the molecular probe was performed using the GoldMagTM-CS kit (Xi’an Gold magnetic nano biotechnology company, GNK0202, Xi’an, China) according to operating instructions. The concentration of HPA antibody is 1 mg/ml. An atomic force micrograph (AFM) was used to evaluate the shape, size, and surface appearance of the nanoparticles. The non-labeled magnetic gold nanoparticles or labeled magnetic gold nanoparticles were placed on the coverslip and were air dried at room temperature for 24 h. The dried samples were analyzed using an atomic force microscope (AFM, Nanoscope, Digital Instruments, Santa Barbara, CA).

### Immunofluorescence

Briefly, cells were cultured on sterile coverslips at 37 °C in a 5% CO_2_ incubator for 48 h. Then, cells were fixed with 4% (*w*/*v*) formaldehyde in PBS for 30 min and permeabilized with 0.2% (*v*/*v*) Triton X-100 for 5 min at room temperature. Cells were blocked by incubating the coverslips with 10% (*v*/*v*) goat serum in PBS for 30 min. Then, cells were stained with HPA&GoldMag molecular probes or normal mouse IgG&GoldMag probes at a dilution of 1:50 followed by Cy3-labeled goat anti-mouse IgG at a 1:100 dilution. The cells were then stained with 0.4 mg/mL DAPI (Sigma-Aldrich) for 10 min at room temperature. Microscopic images were acquired using a Laser Confocal Microscope. Normal mouse IgG&GoldMag probes were used as a negative control.

### Flow Cytometry

The activity of the HPA&GoldMag probe was determined by flow cytometry. Cells were blocked with goat serum for 1 h. Then, the cells were stained with a 10-μg HPA&GoldMag probe or normal mouse IgG&GoldMag at 4 °C overnight, washed four times with PBS, and then incubated with a FITC-labeled antibody against mouse IgG for 1 h at 37 °C. The cells were washed with PBS, and the fluorescence intensity was measured with a flow cytometer (Becton Dickinson).

### In Vitro Study of Magnetic Gold Nanoparticles for MR Imaging

The magnetic gold nanoparticle solution (5 mg/ml) was serially diluted two times (1:20–1:1280) using a 1% agarose solution. The solutions were solidified in EP tubes. MR scanning was performed using a 1% agarose gel as a blank control. The scanning parameters were as follows: T1WI; TR600 ms/TE12 ms; thickness, 2.0 mm; FOV, 150 mm; and T2WI; TR6000 ms/TE92 ms; thickness, 2.0 mm; FOV, 150 mm. HPA&GoldMag MR molecular probes were constructed using HPA mAbs labeled with 30 nm magnetic gold particles. Cells were routinely cultured in 100-mm culture dishes, and HPA&GoldMag probes or negative IgG&GoldMag probes were then added to the cell cultures and incubated at 37 °C for 90 min. Unbound probes were blocked by adding an appropriate amount of goat serum. After washing with PBS three times, cells were digested with trypsin. Cells were then collected, mixed with a 1% agarose solution, and transferred into 1.5-ml EP tubes. MR scanning was performed using a 3.0 T MRI scanner (scanning parameters were the same as described above) using the specific magnetic resonance coil for animals. Before scanning, the mice were anesthetized by 1% pentobarbital and placed in scan bed.

### In Vivo Study of Magnetic Gold Nanoparticles for MR Imaging

Four- to 6-week-old male nude mice (26–30 g) were injected subcutaneously in the hip with 200 μl of MKN45 cells. Each nude mouse was injected with approximately 2.0 × 10^6^ cells. After 2 weeks, the tumors are visible and used for MR imaging. Before injection and 2 h after injection, MR scanning was performed using a 3.0 T MRI scanner. The parameters were T2WI, TR6000 ms/TE92 ms; thickness, 1.5 mm; and FOV, 120 mm. Thereafter, the tumor, spleen, liver, kidney, spleen, heart, and lung tissues were collected to perform Prussian blue Fe staining.

### Statistical Analysis

All the data are presented as the mean ± standard deviation. ANOVA analysis was performed using SPSS13.0 software. A *P* value <  0.05 indicated that the difference was significant.

## Results

### HPA Was Differently Expressed in Various Cancer Cells

**Fig. 1:**
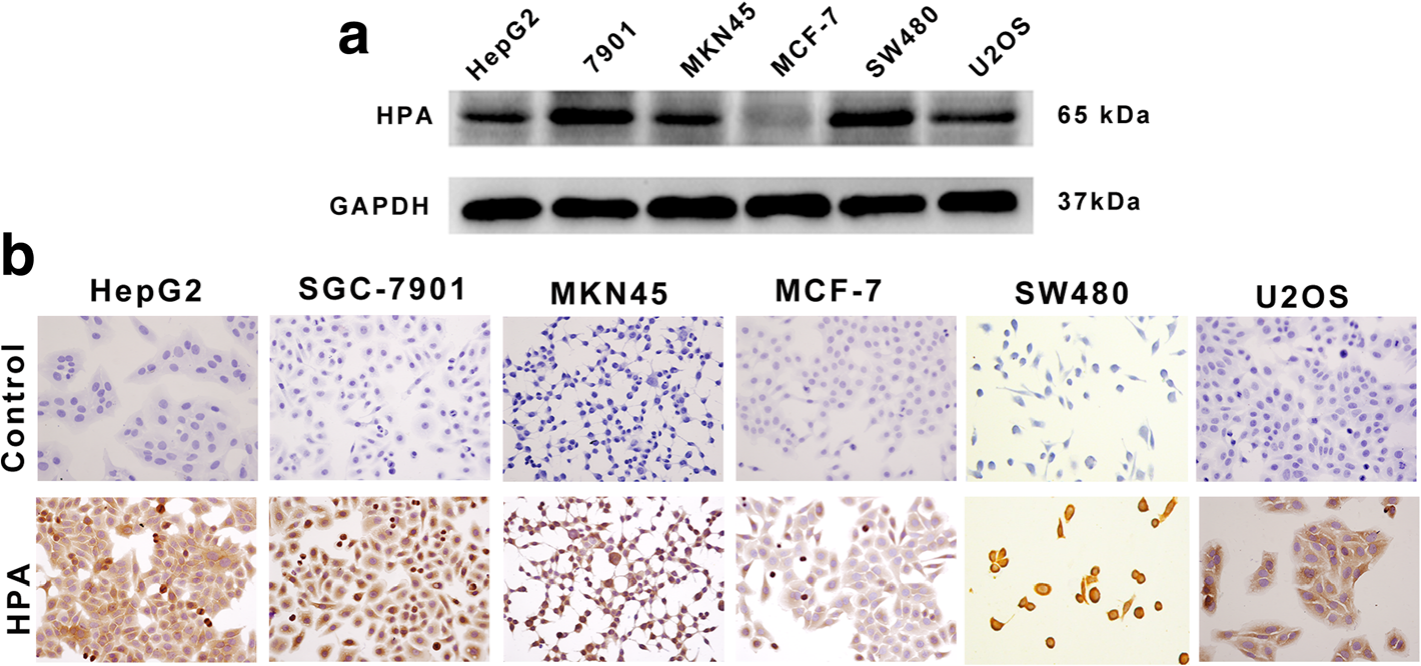
Expression of Hpa proteins in various cell lines. **a** Western blot was used to detect HPA protein expression (65 kDa) in various tumor cell lines. Lane 1, HepG2; lane 2, SGC-7901; lane 3, MKN45; lane 4, MCF-7; lane 5, SW480; lane 5, U2OS. **b** Immunohistochemical analysis of HPA expression in HepG2, SGC-7901, MKN45, MCF-7, SW480, and U2OS cell lines

Both Western blot and immunohistochemical staining analyses were used to test the expression of Hpa in HepG2, SGC-7901, MKN45, MCF-7, SW480, and U2OS cells. The results showed that Hpa expression was much higher in HepG2, SGC-7901, MKN45, SW480, and U2OS cells, whereas much lower expression of Hpa was detected in MCF-7 cells (Fig. 1).

### Construction and Detection of HPA&GoldMag Molecular Probe

**Fig. 2:**
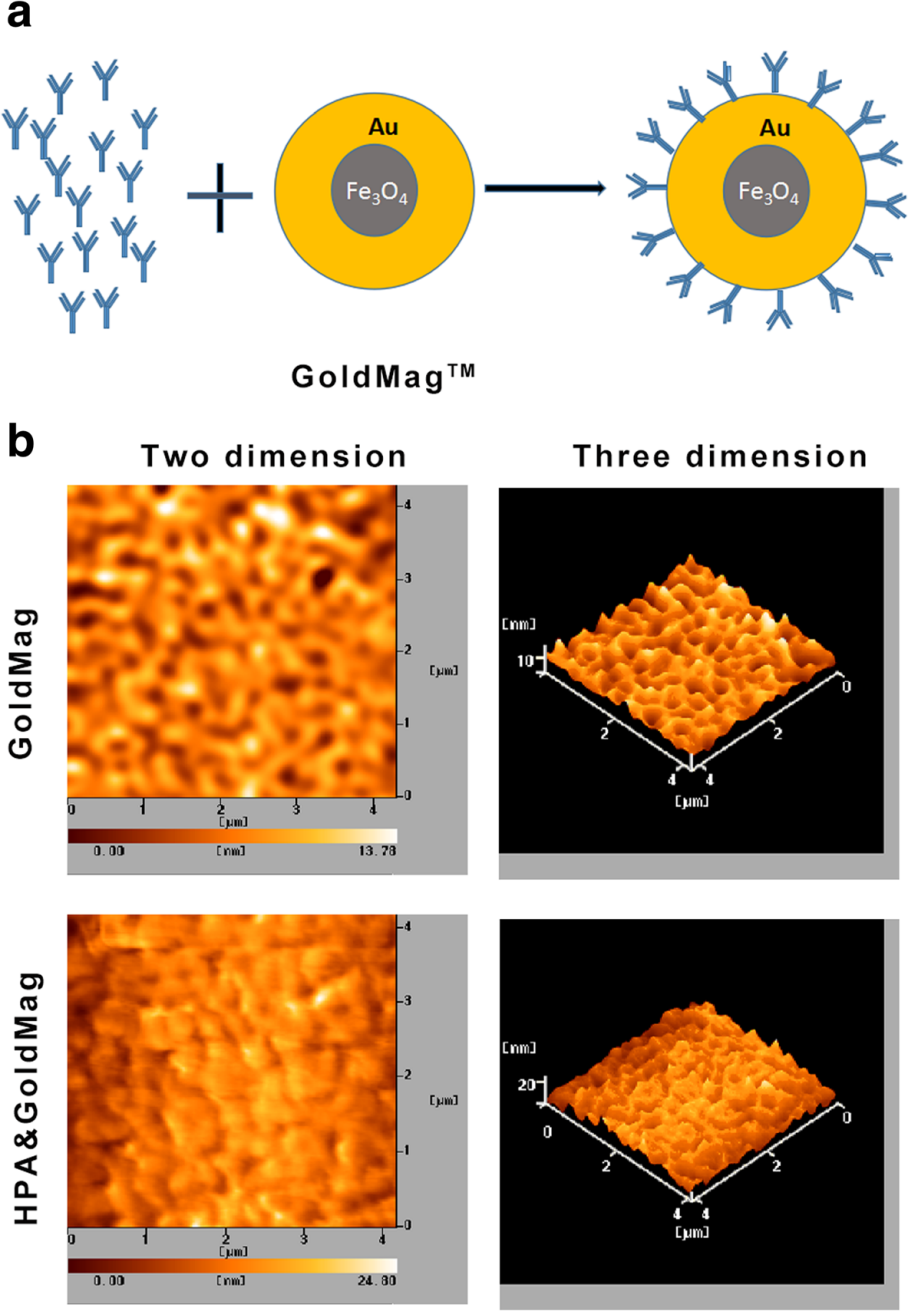
**a** Model of coupling magnetic gold nanoparticles with HPA mAbs. **b** Atomic force scanning of magnetic gold nanoparticles

The HPA&GoldMag molecular probe was prepared by coupling the HPA mAbs with magnetic gold nanoparticles using the coupling reaction between the surfaces of magnetic gold nanoparticles. Atomic force microscopy (AFM) was used to directly observe the surface structure of the probe. We showed that the average diameter of magnetic gold nanoparticles was 13.78 nm without labeling with HPA mAbs (Fig. 2a); the particle sizes were homogenous. After being labeled with HPA mAbs, the average diameter was approximately 24.80 nm (Fig. 2b). These results suggested that magnetic gold nanoparticles were suitable for coupling with HPA mAbs.

### HPA&GoldMag Molecular Probes Can Specifically Bind to HPA

**Fig. 3:**
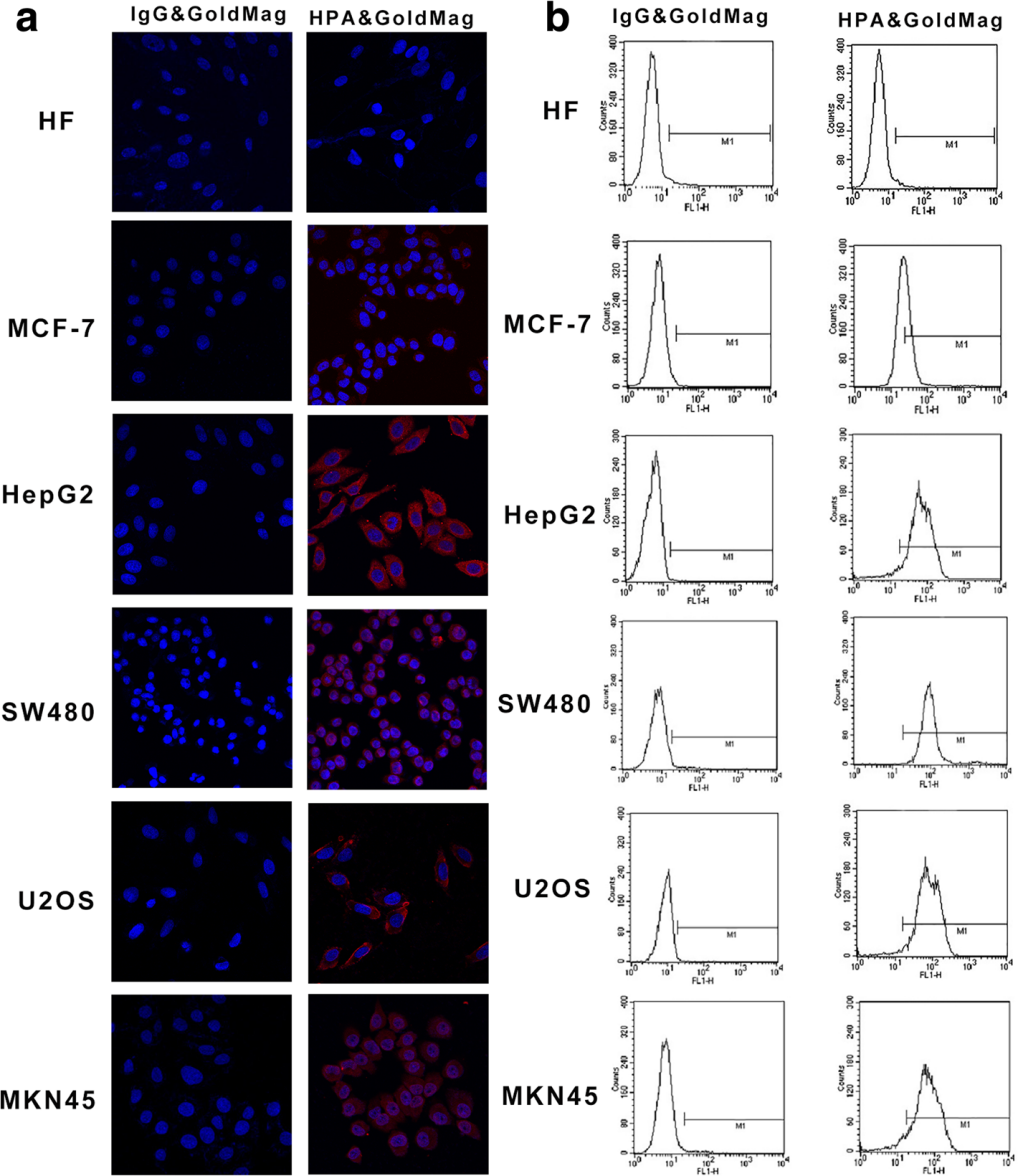
Specificity and binding activity of the probe detected by immunofluorescence and flow cytometry. **a** Immunofluorescence was performed using probes as indicated. **b** Flow cytometry was used to test the specificity of the HPA&GoldMag molecular probe

First, the specificity of the binding between the molecular probe and HPA was evaluated by immunofluorescence. The results showed that a large amount of red fluorescence was detected in the cytoplasm of HepG2, MKN45, SW480, and U2OS cells, while only a small amount of red fluorescence was detected in MCF-7 cells, and no fluorescence was detected in HF cells. However, negative mouse IgG&GoldMag did not show any interaction with HPA in any cell lines (Fig. 3). We further used flow cytometry to test the specificity of the HPA&GoldMag molecular probe. We showed a negative response in HF cells and observed 40% positive rates in MCF-7 cells and 95% positive rates in HepG2, SW480, U2OS, and MKN45 cells. These results indicate that the probes can specifically bind to HPA expressed in tumor cells.

### MR Imaging of HPA&GoldMag Probes In Vitro

**Fig. 4:**
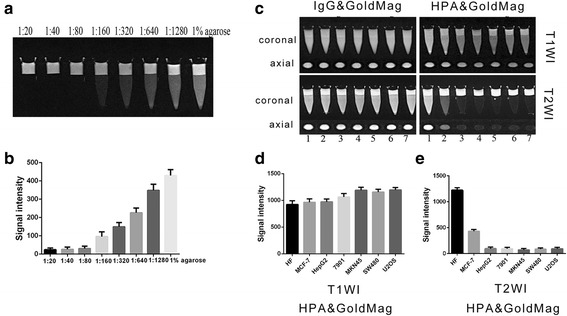
**a** MR imaging of magnetic gold nanoparticles after two serial dilutions. **b** Diagram of the statistical results of MR imaging signal strengths of two serially diluted magnetic gold nanoparticles. **c** MR imaging of all cells after labeling with the probes. Lane 1, HF; lane 2, MCF-7; lane 3, HepG2; lane 4, SGC-7901; lane 5, MKN45; lane 5, SW480; lane 6, U2OS. **d** Statistical results comparing the signal strengths from MR imaging using the T1WI sequence on cells labeled with HPA&GoldMag probes. **e** Statistical results for the signal strengths detected with MR imaging using the T2WI sequence on cells labeled with HPA&GoldMag probes

After serial dilution using 1% agarose, MR imaging of magnetic gold nanoparticles using a T2WI sequence showed different signal reduction. The T2WI signal of a 1:640 dilution was much lower than that of the 1% agarose gel control (*P* < 0.05) (Fig. 4a, b). The results showed that the magnetic gold nanoparticles could reduce the MR signal effectively even in a low concentration, suggesting that magnetic gold nanoparticles could be suitable for molecular imaging. Then, HPA&GoldMag molecular probes were used to label HepG2, SGC7901, MKN45, SW480, U2OS, HF, and MCF-7 cells that were then mixed with 1% agarose and placed under 3.0 T magnetic resonance (MR) (Fig. 4c). MR scanning using T1WI (Fig. 4c, d) or T2WI (Fig. 4c, e) sequence for axial plus coronal scanning. The results showed that compared to T1WI sequence, the signal using T2WI sequence of MR scanning was significantly reduced (*P* < 0.05) while the signal in the HF cells after labeling was not significantly changed (*P* > 0.05), and the signal in the MCF-7 cells after labeling was minimally reduced (*P* < 0.05).

### MR Imaging of HPA&GoldMag Probes in Nude Mice

**Fig. 5:**
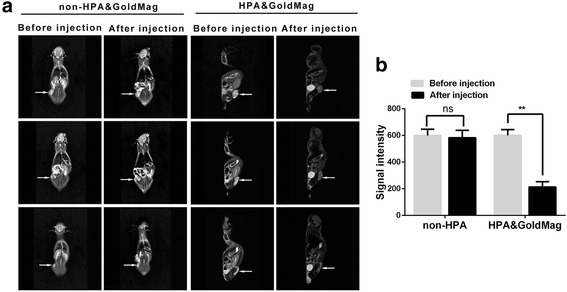
**a** MR imaging of nude mice before and 2 h after the injection of control or HPA&GoldMag probes. The arrow indicates the tumors in mice. **b** Comparison of the signal strengths from MR images using T2WI before and after the injection of the control or HPA&GoldMag probes in nude mice. ^*^P < 0.01

All nude mice were anesthetized with pentobarbital sodium at a dose of 50 mg/kg. The molecular probe was injected into the tail vein of the nude mice, and the MR scan was performed before and 2 h after administration. Since the probe could be absorbed by macrophages in the lung and liver and excreted by the kidney, the scanning results showed that after injection, the tumor, liver, kidney, and lung tissues in the nude mice had significantly reduced signals, compared to the signals that were detected before the injection (*P <* 0.05) (Fig. 5).

## Discussion

Tumor invasion and metastasis is a complex biological process that causes the poor prognosis of tumors. Thus, early diagnosis of tumor metastasis is an important strategy for selecting clinical treatment strategies. MRI is a useful non-invasive method for tumor detection; it enables multidimensional high-contrast tissue imaging. However, MRI could not detect tumors with a small size and high-contrast agents such as metal nanoparticles (iron, gold, etc.) should be used for enhancement of tumor visualization [18–20]. The development of new contrast agents based on new nanomaterials is beneficial to improve MRI techniques for the early detection of cancers. Among the imaging agents, the superparamagnetic iron oxide particle (SPIO) is used as a common contrast agent in MR molecular imaging, in which magnetic gold nanoparticles are superparamagnetic nanocomposite materials with a Fe_3_O_4_ (cores)/Au (shell) structure [21–24]. The superparamagnetic properties of inorganic nanoparticles are useful for magnetic resonance imaging (MRI) [25]. A combination of these nanoparticles with tissue-specific agents (antibodies or low-molecular weight substances) enables precise detection and diagnosis of tumors. Numerous studies have shown that HPA is expressed in many tumors, and its expression level is closely associated with the metastatic potential of tumors [26]. HPA can destroy the integrity of the ECM and BM through the degradation of HSPG, which releases active molecules such as bFGF, HGF, and VEGF anchored in the ECM, thus promoting tumor progression [27–30]. Since HPA is mainly expressed in mid- to late-stage tumors, our previous studies have shown that HPA could be used as a common TAA for tumor therapy [13–15]. Therefore, HPA might be a potentially useful target for tumor molecular imaging and therapy.

Magnetic gold nanoparticles are superparamagnetic composite nanomaterials with a Fe_3_O_4_ (core)/Au (shell) structure that are produced by the reduction of Au^3+^ by hydroxylamine hydrochloride from superparamagnetic Fe_3_O_4_ particles [31, 32]. Since the labeling method is quite simple, they can be used for biological applications such as cell selection, protein purification, nucleic acid separation, and immunoassays. Hence, by following an established procedure, we were able to successfully prepare and characterize gold-coated iron oxide nanoparticles linked to anti-HPA antibodies. In this research, 30-nm magnetic gold particles were used because they are much more stable than 50-nm magnetic gold particles. One milligram of 30-nm magnetic gold particles could bind to approximately 200 μg of HPA mAbs. Our data showed that the use of magnetic gold nanoparticles at a 1:640 dilution (approximate 10 μg) produced reduced signaling compared to the blank control group in 3.0 T MRI scanning (*P <* 0.05). Thus, the critical value for in vivo imaging using magnetic gold particles could be calculated.

The activity and specificity of the magnetic gold nanoparticle-coupled HPA mAb probes was subsequently detected using laser confocal microscopy, flow cytometry, and atomic force microscopy. Briefly, the probe was tested in vitro by incubation of the targeted probe and the negative control probe with both HPA (+) and (−) cells. Here, human MCF-7 breast cancer cells displayed weak HPA expression, while MKN45, SW480, U2OS, HepG2, and SGC-7901 cells had higher HPA expression. Furthermore, normal mouse IgG was used as a control for the HPA mAbs to further prove the specificity of the nanoparticle.

After the specificity of the probe was tested in vitro*,* the targeted and control probes were injected in nude mice subcutaneously injected with human MKN45 gastric cancer cells. Once the diameter of the tumor tissues reached 1 cm, MR scanning was performed using a 3.0 T MRI chamber to detect signal changes in tumors before and after the injection with probes. The scanning results showed that tumor signals were significantly reduced 2 h after probe injection compared to the signals before probe injection. These results demonstrated that the HPA&GoldMag probes had excellent immune activities and better effects in nude mice bearing MKN45 cells.

## Conclusions

In summary, we demonstrated that the HPA&GoldMag molecular probes coupled with HPA mAbs had excellent physical and chemical properties. The probe could specifically target many tumor cells expressing high HPA both in vitro and in vivo. Using 3.0 T MRI scanning, the probes were shown to significantly reduce the T2WI signal in tumor tissues; this may provide an experimental base for molecular imaging of tumor metastasis.

## Abbreviations

AFM: Atomic force micrograph
BM: Basement membrane
CT: Computed tomography
ECM: Extracellular matrix
HPA: Heparanase
HSPG: Heparan sulfate proteoglycan
MRI: Magnetic resonance imaging
PVDF: Polyvinylidenedifluoride
TAA: Tumor-associated antigen
